# A new prognostic nomogram in patients with mucosa-associated lymphoid tissue lymphoma: a multicenter retrospective study

**DOI:** 10.3389/fonc.2023.1123469

**Published:** 2023-04-25

**Authors:** Qiuyue Wen, Xiaoqian Li, Kewei Zhao, Qiuhui Li, Fang Zhu, Gang Wu, Tongyu Lin, Liling Zhang

**Affiliations:** ^1^ Cancer Center, Union Hospital, Tongji Medical College, Huazhong University of Science and Technology, Wuhan, China; ^2^ Department of Medical Oncology, Shandong Cancer Hospital, Shandong Academy of Medical Sciences, Jinan, China; ^3^ Department of Medical Oncology, Sichuan Cancer Hospital and Institute, Sichuan Cancer Center, School of Medicine University of Electronic Science & Technology of China, Sichuan, Chengdu, China; ^4^ Department of Medical Oncology, Sun Yat-sen University Cancer Center, Guangzhou, State Key Laboratory of Oncology in Southern China, and Collaborative Innovation Center of Cancer Medicine, Guangzhou, China

**Keywords:** MALT lymphoma, prognosis, nomogram, inflammatory markers, therapy

## Abstract

**Background:**

The present study sought to understand how clinical factors and inflammatory biomarkers affected the prognosis of mucosa-associated lymphoid tissue (MALT) lymphoma and develop a predictive nomogram to assist in clinical practice.

**Methods:**

We conducted a retrospective study on 183 cases of newly diagnosed MALT lymphoma from January 2011 to October 2021, randomly divided into two groups: a training cohort (75%); and a validation cohort (25%). The least absolute shrinkage and selection operator (LASSO) regression analysis was combined with multivariate Cox regression analysis to construct a nomogram for predicting the progression-free survival (PFS) in patients with MALT lymphoma. To evaluate the accuracy of the nomogram model, the area under the receiver operating characteristic (ROC) curves, calibration curves, and decision curve analysis (DCA) were used.

**Results:**

The PFS was significantly associated with the Ann Arbor Stage, targeted therapy, radiotherapy, and platelet-to-lymphocyte ratio (PLR) in MALT lymphoma. These four variables were combined to establish a nomogram to predict the PFS rates at three and five years. Importantly, our nomogram yielded good predictive value with area under the ROC curve (AUC) values of 0.841 and 0.763 in the training cohort and 0.860 and 0.879 in the validation cohort for the 3-year and 5-year PFS, respectively. Furthermore, the 3-year and 5-year PFS calibration curves revealed a high degree of consistency between the prediction and the actual probability of relapse. Additionally, DCA demonstrated the net clinical benefit of this nomogram and its ability to identify high-risk patients accurately.

**Conclusion:**

The new nomogram model could accurately predict the prognosis of MALT lymphoma patients and assist clinicians in designing individualized treatments.

## 1 Introduction

It is well-established that marginal zone lymphomas (MZLs) are derived from B cells in the marginal zone of the follicle. Three subtypes of MZLs can be distinguished based on the site of involvement: extranodal mucosa-associated lymphoid tissue marginal zone lymphoma (MALT), splenic marginal zone lymphoma (SMZL) and lymph node marginal zone lymphoma (NMZL) (1). MZL, NMZL, and SMZL reportedly account for approximately 10%, less than 2%, and less than 1% of non-Hodgkin lymphomas (NHLs), respectively. MALT lymphoma is less common than follicular and diffuse large B-cell lymphoma, accounting for about 7–8% of NHLs (2). MALT lymphoma can be divided into gastric MALT and non-gastric MALT lymphoma. According to the World Health Organization classification released in 2008, the stomach is the most specific organ of origin (representing about 50% of MALT lymphoma) (3), followed by the lungs, head and neck, and orbit. Reports of intestines, liver, thyroid, and breast involvement are rare. MALT lymphoma has a predilection age of 50 to 60 years, and the progression is relatively slow. At present, its pathophysiology is not yet entirely understood. Numerous investigations have shown that MALT lymphoma is associated with autoimmune diseases (4), such as the thyroid MALT lymphoma linked to persistent autoimmune thyroiditis. Moreover, helicobacter pylori (HP) infection is present in approximately 90% of patients with gastric MALT lymphoma (5). Pulmonary MALT lymphoma may be associated with Achromobacter xylosoxidans or Chlamydophila psittaci infection (6), which is also associated with ocular adnexal MALT lymphoma. Moreover, intestinal MALT lymphoma may be linked to Campylobacter jejuni (7).

In short, the etiology, clinical manifestations, and treatment methods differ in MALT lymphoma patients. Approximately 75% to 80% of patients with stomach MALT lymphoma who have an HP infection experience remission after HP eradication (8). Notably, t(11,18) chromosome translocation is most common in MALT lymphoma, and anti-HP treatment may be ineffective in this patient population (9). Additional therapies for MALT lymphoma include lenalidomide, radiation, rituximab or combination chemotherapy, surgery alone, and these other therapies (10). According to the US SEER database, MALT lymphoma has a favorable prognosis with a 5-year relative survival rate of 89% (11). However, patients with MALT lymphoma frequently experience recurrence during long-term follow-up (12). The International Prognostic Index (IPI) has been extensively used to analyze patient prognosis for MALT lymphoma (13). However, considering the various factors contributing to the etiology of this rare population, it is necessary to provide new simple and effective prediction tools. Inflammatory biomarkers (neutrophil, platelet, and lymphocyte counts) also play a significant part in the pathogenesis and growth of malignancies (14–16). To our knowledge, no study has reported the association between inflammatory biomarkers and recurrence in MALT lymphoma. Accordingly, this research aimed to investigate which clinical characteristics and inflammatory biomarkers affected MALT lymphoma patients’ prognoses to create a comprehensive nomogram that would be valuable for personalized treatment during clinical practice.

## 2 Materials and methods

### 2.1 Patients

One hundred eighty-three patients diagnosed with MALT lymphoma at three tumor centers (Wuhan Union Hospital, Sun Yat-sen University, and Sichuan Provincial People’s Hospital) were included from January 2011 to October 2021 in this study. 137 MALT patients were randomly attributed to the study cohort and 46 to the validation cohort. The inclusion criteria were as follows (1): histologically confirmed MALT lymphoma according to the WHO Classification of Tumors of Hematopoietic and Lymphoid Tissues; (2) patients received at least one anti-tumor therapy (surgery, radiotherapy, chemotherapy, targeted therapy, and anti-HP therapy) after diagnosis of MALT lymphoma. To be clear, targeted therapy here means targeted therapy (rituximab) combined with or without chemotherapy; (3) The included patients completed the follow-up and complete clinical data was available. This study was approved by the Ethics Committees of Cancer Center, Union Hospital, Tongji Medical College, and Huazhong University of Science and Technology. The institutional review committees of each cooperating agency also approved the initiative.

### 2.2 Data collection and follow-up

We collected the clinical data of MALT lymphoma patients, including the gender, age, Ann Arbor stage, lactate dehydrogenase (LDH), Globulin, IPI, Ki67 level, B symptoms, Eastern Cooperative Oncology Group Performance Status (ECOG PS), hepatitis B surface antigen status at diagnosis, number and site of extranodal involvement, plasmacytic differentiation (PCD), white blood cell (WBC), hemoglobin (Hb), lymphocytes, platelet (PLT), neutrophils, monocytes, systemic immune-inflammation index (SII), platelet-to-lymphocyte ratio (PLR), neutrophil-to-lymphocyte ratio (NLR), lymphocyte-to-monocyte ratio (LMR), and anti-tumor treatment regimen. Treatment options include radiotherapy, surgery, chemotherapy, and targeted therapy. In some cases, anti-HP treatment was provided. The prognostic indicators mentioned above were based on recommendations of guidelines or published in earlier studies. The absolute platelet count was multiplied by the absolute neutrophil count (ANC) to lymphocyte count (ALC) ratio to determine the SII. The NLR was calculated by dividing the ANC by the absolute lymphocyte count. The LMR was the proportion of monocyte count to ALC. The PLR was the proportion of the absolute platelet count to the ALC. Each patient underwent Ann Arbor staging according to their condition. The progression-free survival (PFS) was measured at the final follow-up. It was the period between the diagnosis and the progression of the disease, its recurrence, or the patient’s death from any cause.

### 2.3 Construction of nomogram and statistical analysis

The continuous variables were expressed by the median, while the categorical variables were expressed by the frequency and percentage (%). A chi-square test was performed to assess the differences between the training and validation groups. The training cohorts were first examined for prospective prognostic factors to establish the nomogram using LASSO regression (17). This approach made it possible to choose the variables while estimating the model’s parameters, which could better deal with the issue of multiple commonalities in regression analysis. The independent prognostic risk factors related to MALT lymphoma were established using a multivariate Cox proportional hazards model. The endpoint of the study was the PFS. The objects analyzed included all of the above factors. Each variable was specified as an input with a taxonomic or binary type. In this study, we constructed the nomogram based on independent prognostic variables and determined the total score based on each patient. Finally, the time-dependent receiver operating characteristic curve (ROC) of these two groups was constructed to verify the accuracy of our nomogram. The predicted and actual prognoses were contrasted visually using the calibration charts. This nomogram’s clinical value and applicability were evaluated using Decision Curve Analysis (DCA) (18). The threshold probability served as the DCA’s abscissa, while the ordinate, or net profit rate, was obtained after subtracting benefits and drawbacks. Kaplan -Meier curves were used to assess the progression-free survival of high-risk and low-risk populations discriminated by nomogram. For the statistical analysis in this work, the SPSS statistical program (version 27.0; IBM Corporation; Armonk, NY), Empower Stats (www.empowerstats.com) and R software (version 4.2.0) were utilized. The hazard ratio (HR) and the corresponding 95% credible interval (CI) were calculated, and two-sided P values<0.05 were statistically significant.

## 3 Results

### 3.1 Clinical characteristics

In our study, 183 patients met the inclusion criteria and were randomly separated into training (n=137) and validation (n=46) cohorts. **Table 1** displays the clinical characteristics of the training and validation groups. The mean age at disease onset of the training cohort was 54 (range 15-78), with a female predominance (60.6%). Most patients (95.6%) had an ECOG PS score of 0 to 1. Stage III or IV patients represented 8.0%. According to the IPI, 131 (95.6%) patients were at low risk. Patients with and without stomach involvement as primary sites accounted for 33.6% (n=46) and 66.4% (n=91) of cases, respectively. 37.2% and 29.2% of patients in the training dataset received chemotherapy and targeted therapy, respectively. Moreover, patients who received surgery and radiotherapy accounted for 49.6% and 59.1%, respectively. During the 33-month median follow-up period, the overall recurrence rate was 16.1% (22/137).

**Table 1:** Clinical characteristics of patients.

Characteristics	Training cohort (%) (n = 137)	External validation cohort (%) (n = 46)
Age		
≤54	75 (54.7)	17 (37.0)
>54	62 (45.3)	29 (63.0)
Sex		
Male	54 (39.4)	21 (45.7)
Female	83 (60.6)	25 (54.3)
ECOG PS		
0-1	131 (95.6)	42 (91.3)
>1	6 (4.4)	4 (8.7)
B symptom		
No	133 (97.1)	45 (97.8)
Yes	4 (2.9)	1 (2.2)
Ann Arbor stage		
I-II	126 (72.4)	10 (21.7)
III-IV	11 (8.0)	36 (78.3)
Primary site		
Gastric	46 (33.6)	13 (28.3)
Non-gastric	91 (66.4)	33 (71.7)
Surgery		
No	69 (50.3)	22 (47.8)
Yes	68 (49.6)	24 (52.2)
Chemotherapy		
No	86 (62.8)	11 (23.9)
Yes	51 (37.2)	35 (76.1)
Targeted therapy		
No	97 (70.8)	19 (41.3)
Yes	40 (29.2)	27 (58.7)
Radiotherapy		
No	56 (40.9)	27 (58.7)
Yes	81 (59.1)	19 (41.3)
Number of extranodal involvement		
≤1	121 (88.3)	15 (32.6)
>1	16 (11.7)	31 (67.4)
PCD		
No	109 (79.6)	35 (76.1)
Yes	28 (20.4)	11 (23.9)
HBsAg status		
No	121 (88.3)	36 (78.3)
Yes	16 (11.7)	10 (21.7)
IPI		
0-1	131 (95.6)	10 (21.7)
>1	6 (4.4)	36 (78.3)
WBC (10^9^/L)		
≤5.06	69 (50.4)	23 (50.0)
>5.06	68 (49.6)	23 (50.0)
Hemoglobin (g/L)		
≤128	72 (52.6)	24 (52.2)
>128	65 (47.4)	22 (47.8)
PLT (10^9^/L)		
≤201	69 (50.4)	27 (58.7)
>201	68 (49.6)	19 (41.3)
Lymphocytes (10^9^/L)		
≤1.46	80 (58.4)	29 (63.0)
>1.46	57 (41.6)	17 (37.0)
Neutrophils (10^9^/L)		
≤2.88	69 (50.4)	22 (47.8)
>2.88	68 (49.6)	32 (69.6)
Monocytes (10^9^/L)		
≤0.35	71 (51.8)	16 (34.8)
>0.35	66 (48.2)	30 (65.2)
NLR		
≤2.01	68 (49.6)	15 (32.6)
>2.01	69 (50.4)	31 (67.4)
PLR		
≤131.47	69 (50.4)	21 (45.7)
>131.47	68 (49.6)	25 (54.3)
LMR		
≤4.13	68 (49.6)	31 (67.4)
>4.13	69 (50.4)	15 (32.6)
SII		
≤402.31	69 (50.4)	18 (39.1)
>402.31	68 (49.6)	28 (60.9)
Globulin (g/L)		
≤27.9	70 (51.1)	24 (52.2)
>27.9	67 (48.9)	22 (47.8)
LDH (U/L)		
≤160	69 (50.4)	17 (37.0)
>160	68 (49.6)	29 (63.0)

ECOG PS, Eastern Cooperative Oncology Group performance status; PCD, plasmacytic differentiation; HBsAg status, HBsAg, Hepatitis B virus surface antigen; WBC, white blood cell; PLT, platelet; LDH, lactic dehydrogenase; LMR, lymphocyte- to- monocyte ratio; NLR, neutrophil-to-lymphocyte ratio; PLR, platelet-to-lymphocyte ratio; SII, systemic immune-inflammation index; LDH, lactic dehydrogenase.

### 3.2 Identifying independent prognostic factors for PFS

**Figure 1:**
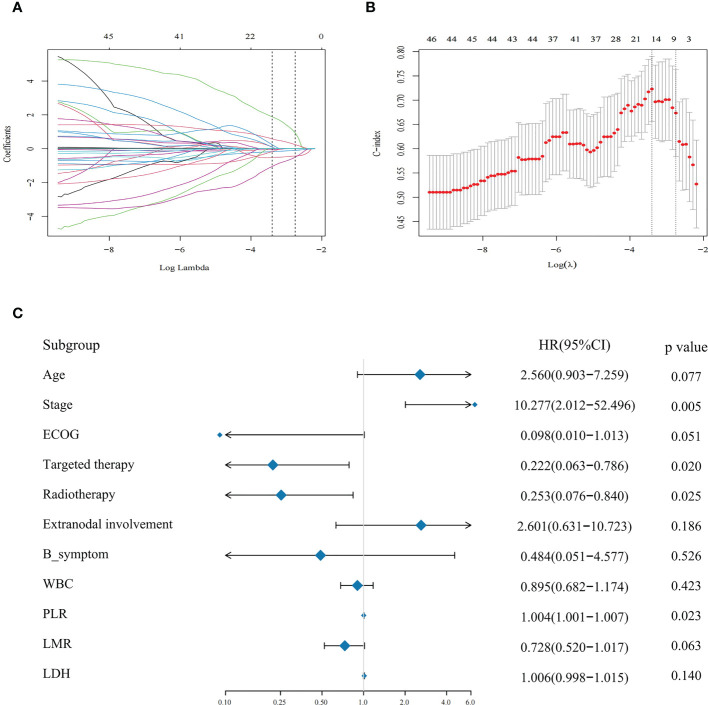
**(A, B)** Eleven potential prognostic factors were screened using LASSO analysis with minimal lambda. **(C)** COX regression analysis screened four independent influencing factors for PFS and presented them in a forest plot.

The median PFS was used to separate patients into a high and low-risk groups. In the training cohort, the above indices were analyzed by LASSO regression analysis to screen for prognostic factors affecting the relapse of MALT lymphoma (**Figures 1A, B**). Our findings revealed that age, Ann Arbor stage, LDH, B symptoms, ECOG PS, radiotherapy, targeted therapy, number of extranodal involvement, WBC, PLR, and LMR were contributing factors for PFS. The above parameters were incorporated in multivariate Cox regression analysis to screen out the independent influencing factors of PFS in MALT lymphoma. Four factors were significantly associated with PFS (**Figure 1C**). Among them, radiotherapy (HR = 0.253, 95% CI = 0.076-0.840, *P* = 0.025) and targeted therapy (HR = 0.222, 95% CI = 0.063-0.786, *P* = 0.020) were independent predictors in people with MALT lymphoma and could reduce recurrence. Patients with a high PLR (HR = 1.004, 95% CI = 1.001-1.007, *P* = 0.023) and Ann Arbor III or IV stage (HR = 0.005, 95% CI = 2.012-52.496, *P* = 0.005) were risk factors associated with patient relapse.

### 3.3 Nomogram construction and validation

**Figure 2:**
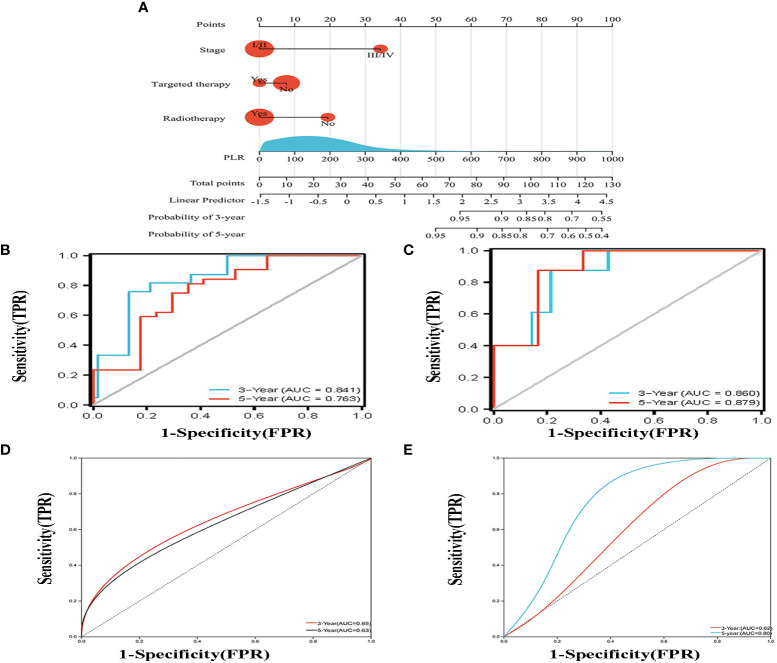
**(A)** Nomogram for predicting the 3- and 5-year PFS probability for patients with MALT lymphoma. The nomogram includes a total risk score and clinical characteristics. **(B)** Progression-free survival probability curves were plotted against two risk groups defined by the nomogram in the training cohort. **(C)** According to the drawn nomogram, the progress-free survival probability of the validation cohort patients was calculated. **(D**, **E)** Progression-free survival curves were drawn for the training cohort and the validation cohort based on the MALT-IPI score, respectively.

**Figure 3:**
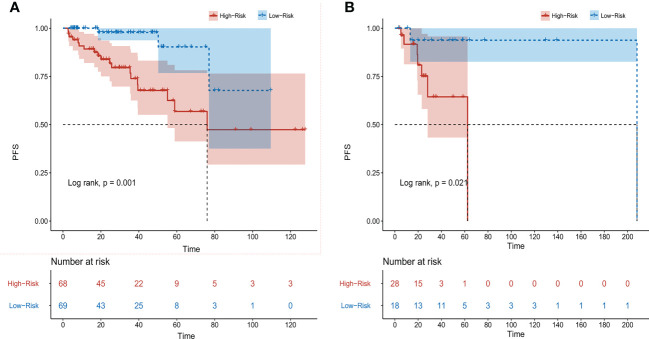
Progression-free survival (PFS) of patients with MALT lymphoma was analyzed based on nomogram risk stratification. **(A)** Training cohort patients. **(B)** Validation cohort patients.

**Figure 4:**
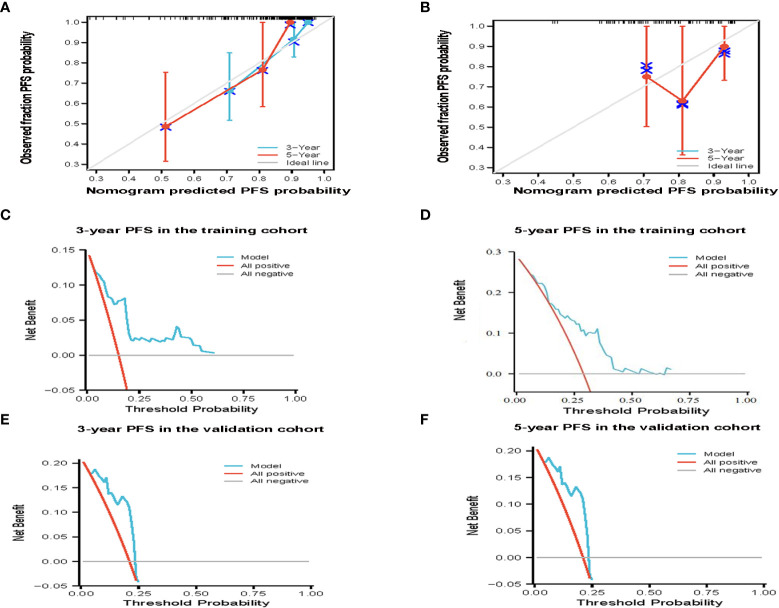
The effectiveness of the constructed risk prediction model. Calibration curves of the nomogram at 3-year and 5-year PFS in the training cohort **(A)** and validation cohort patients **(B)**. Prediction of the 3-year and 5-year PFS in patients with MALT lymphoma by decision curve analysis based on nomogram in the training cohort **(C, D)** and validation cohort patients **(E, F)**.

According to the results of multivariate analysis, a nomogram based on the four prognostic factors (Ann Arbor stage, targeted therapy, radiotherapy, and PLR) was established and could predict the 3-year and 5-year PFS (**Figure 2A**). Each independent risk factor in the nomogram corresponded to a specific score, and each variable was calculated and merged. Patients with high scores indicated that they had an increased risk of recurrence. Then, using Kaplan-Meier (KM) curves analysis, events were split into high-risk and low-risk groups, with the high-risk group having a higher likelihood of relapsing (P = 0.001) (**Figure 3A**). The AUC values for the 3-year and 5-year PFS were 0.841 and 0.763, respectively (**Figure 2B**). The prognostic ROC curves of the nomogram and MALT-IPI in this study showed that the areas under the 3 and 5-year recurrence rates according to the nomogram were better than those under the MALT-IPI (0.650 and 0.630) (**Figure 2D**). The nomogram’s calibration curve demonstrated high concordance between the predicted 3- and 5-year PFS and the actual PFS (**Figure 4A**).

The PFS nomogram subsequently underwent external validation. The AUC values for the 3- and 5-year PFS and calibration plots showed consistency between the training and validation cohorts (**Figures 2C**, **4B**). The ROC curve for the validation cohort also showed that the new nomogram measuring 3-year and 5-year PFS had better sensitivity and specificity than MALT-IPI (**Figure 2E**). In addition, the validation cohort from the nomogram anticipated the total score, which was divided into high-risk and low-risk. The Kaplan-Meier curve revealed a higher recurrence rate in high-risk populations (P = 0.021) (**Figure 3B**). As demonstrated by DCA, the model provided a net clinical benefit (**Figures 4C–F**). Our nomogram could accurately predict 3- and 5-year PFS in MALT lymphoma.

Then, we further compared the PFS between groups classified according to the MALT-IPI. However, in PFS analysis, MALT-IPI could not distinguish between low-risk and medium-risk groups (**Figure S1**).

## 4 Discussion

In the present study, we found that the Ann Arbor stage, targeted therapy, radiation therapy, and PLR were significant factors that affected the PFS of patients with MALT lymphoma using LASSO and Cox analyses. A prognostic model was established for risk stratification of this patient population. Moreover, we demonstrated the model’s accuracy, which underwent internal and external validation. To our knowledge, this is the first study to report a prognostic model based on inflammatory indicators for MALT lymphoma patients.

It has been established that MALT lymphoma can occur in all organs. The stomach is the most common organ involved, accounting for 35% of all extranodal lymphomas. Compared with other intranodal lymphomas, MALT lymphoma has distinct histological, immunophenotypic, and genotypic characteristics, with significantly different clinical manifestations and prognoses. It is mainly characterized by slow progression, low invasiveness, and an indolent clinical process. It can transform to highly malignant large B-cell lymphoma in some cases with an incidence of less than 10%. The current treatment methods include radiotherapy, chemotherapy, rituximab combined with chemotherapy, surgical resection of the tumor, antibiotics, and various integrated treatments. The International Prognostic Index (IPI) based on patient age, extranodal lesions, Ann Arbor stage, ECOG PS, and LDH value can distinguish patients with different prognoses and widely used in aggressive lymphoma. MALT-IPI (Age, LDH, and Ann Arbor stage)was conducted to predict the prognosis in 2017. However, the results of peripheral blood tests related to cancer treatment have not been included. Moreover, studies have shown that inflammatory cells and cytokines in tumors have a higher propensity to promote tumorigenesis, development, and immunosuppression than to produce a potent host anti-tumor response. Besides, clinical evidence shows a direct relationship between chronic inflammation and tumors. Blocking transforming factor TGF- β, for example, can lead to neutrophil depletion, reducing the anti-tumor effect of treatment (19). There is a growing consensus suggesting that platelets are recruited to wrap tumor cells to protect them from immune responses and promote the growth and spread of cancer (20, 21). Tumor-associated macrophages (TAM) are produced from circulating monocyte progenitors, which means that monocytes can increase tumor cell proliferation, facilitate angiogenesis, and accelerate invasion and metastasis (22). There is rich literature available substantiating the predictive value of inflammatory markers, including PLR, NLR, LMR, and SII in solid tumors (breast tumors, lung cancer, etc.) and diffuse large B cell lymphoma (DLBCL) (23–28). High NLR and SII are poor prognostic factors for tumors. Moreover, low LMR is an adverse prognostic indicator for malignancies. However, the effect of these inflammatory markers on MALT lymphoma has not been reported so far. Therefore, it remains unclear whether they affect the prognosis of MALT lymphoma.

In our study, we collected the laboratory and clinical data of 137 patients with MALT lymphoma and evaluated the impact of PLR, NLR, LMR, SII, and other clinical variables on the prognosis of MALT lymphoma. In addition, due to the long survival times of MALT lymphoma patients, there were only three fatal MALT lymphoma events in this study, making statistical analysis meaningless. Therefore, we focused on the new prognostic model for PFS rather than overall survival (OS).

In the preliminary analysis, in view of the clinical significance and in order to avoid over-fitting of the model, we only included three indicators of MALT IPI. Univariate analysis showed that only Ann Arbor stage had statistical significance for recurrence and the Ann Arbor stage was finally identified as an independent predictor in patients with MALT lymphoma by multivariate analysis. Importantly, tumor staging describes the severity and scope of involvement of malignant tumors according to the degree of the primary tumor and its spread. Unlike other malignant tumors, the stage of lymphoma is determined according to the extent of the tumor’s distribution. The Ann Arbor stage is a widely recognized classification standard for lymphoma used as a prognostic indicator for most lymphoma subtypes. Moreover, it has been widely used in MALT lymphoma. The recurrence rate of MALT lymphoma during the early stage is lower than during the late stage, which has been confirmed in our study. Different treatments have different prognoses, so some studies have incorporated the treatment model of patients into the prognostic model (29–33). Our study also found that radiotherapy was an independent factor affecting the PFS in MALT lymphoma. In this respect, it has been reported that radiotherapy has been utilized as a local treatment for patients with an early stage of MALT lymphoma to achieve long-term disease control (34, 35). Radiotherapy is also a good rescue approach for patients with relapses. Recently, much emphasis has been placed on RT downgrade to avoid treatment-related morbidity (36). At present, targeted therapy plays a significant role in cancer treatment. Numerous prospective studies on targeted therapy alone and paired with chemotherapy have been conducted domestically and internationally (37). When administered as first-line therapy to patients with indolent NHL, rituximab yielded promising results (38). According to our study, rituximab combined with chemotherapy significantly decreased the recurrence rate in individuals with MALT lymphoma.

Moreover, we found that PLR was an independent prognostic risk factor. As previously mentioned, lymphocytes can yield a higher immune response and suppress tumorigenesis, whereas platelets can promote tumorigenesis. We assessed PLR’s impact on MALT lymphoma based on the aforementioned findings. Our findings revealed that patients with MALT lymphoma with high PLR had a higher probability of relapsing. High PLR rates in advanced patients were associated with more significant relapses. Patients who underwent radiotherapy and targeted therapy had a decreased risk of recurrence. Importantly, our nomogram effectively distinguished the low-risk group from the high-risk group based on the PFS. Indeed, it is well-established that inflammatory indicators and the tumor microenvironment are tightly connected. Although several studies have observed that systemic inflammatory markers are an essential indicator of prognosis and pre-treatment in other malignancies, no research has explored inflammatory markers of MALT lymphoma. Therefore, we established a new nomogram based on clinical characteristics and inflammatory markers. It is important to note that although the ROC curve was used to assess the model’s accuracy during internal and external validation, false positives and negatives may occur. The DCA curve, on the other hand, may effectively avoid this issue because of the modest requirements for the sample data set and the total independence of the components during processing, allowing it to address multicollinearity in medical statistical analysis. As a result, DCA was included in this study. It has also been demonstrated that our model has substantial returns. Importantly, our nomogram has enormous prospects for assisting clinicians during individualized treatment.

The present study does, however, contain several things that could be improved. First, biases, including selection bias, detection bias, and analytical bias, could have happened because it was a small sample-size retrospective study. Besides, given that the investigation spans multiple centers, testing results may differ between hospitals due to testing chemicals and apparatus differences. Finally, different approaches in treating patients following surgical resection have also been documented in the literature and can result in various clinical outcomes.

## 5 Conclusion

Our new nomogram model has a prospect for clinical application for predicting PFS in patients with MALT lymphoma. However, more large-sample size studies are warranted to validate its clinical value.

## Data availability statement

The original contributions presented in the study are included in the article/**Supplementary Material**. Further inquiries can be directed to the corresponding author.

## Ethics statement

The studies involving human participants were reviewed and approved by Ethical Committees of Union Hospital, Tongji Medical College, Huazhong University of Science and Technology. Written informed consent from the participants’ legal guardian/next of kin was not required to participate in this study in accordance with the national legislation and the institutional requirements.

## Author contributions

Design the study: LZ, QW, and XL; methodology, QW and XL Analysis: QW and XL. Investigation: KZ, QL, and FZ. Data curation: QW, XL, KZ, QL, and FZ. Writing- original draft preparation: QW. Writing-review and editing: QW, XL, and KZ. Supervision: TL and GW. All authors contributed to the article and approved the submitted version.
