# Supplementary material for: A new prognostic nomogram in patients with mucosa-associated lymphoid tissue lymphoma: a multicenter retrospective study
Source: Front Oncol. 2023 Apr 25;13:1123469. doi: 10.3389/fonc.2023.1123469 (PMC10166839; doi:10.3389/fonc.2023.1123469)
Supplement: Supplementary file 1 [file DataSheet_1.docx]

Supplementary Material

**A new prognostic nomogram in patients with mucosa- associated lymphoid tissue lymphoma: A multicenter retrospective study**

Qiuyue Wen^1†^， Xiaoqian Li^2†^, Kewei Zhao^1^, Qiuhui Li^1^, Fang Zhu^1^, Gang Wu^1^, Tongyu Lin^3,4^, Liling Zhang^1*^

*** Correspondence:** Corresponding Author: [lily-1228@hotmail.com](mailto:lily-1228@hotmail.com)

# Supplementary Figure


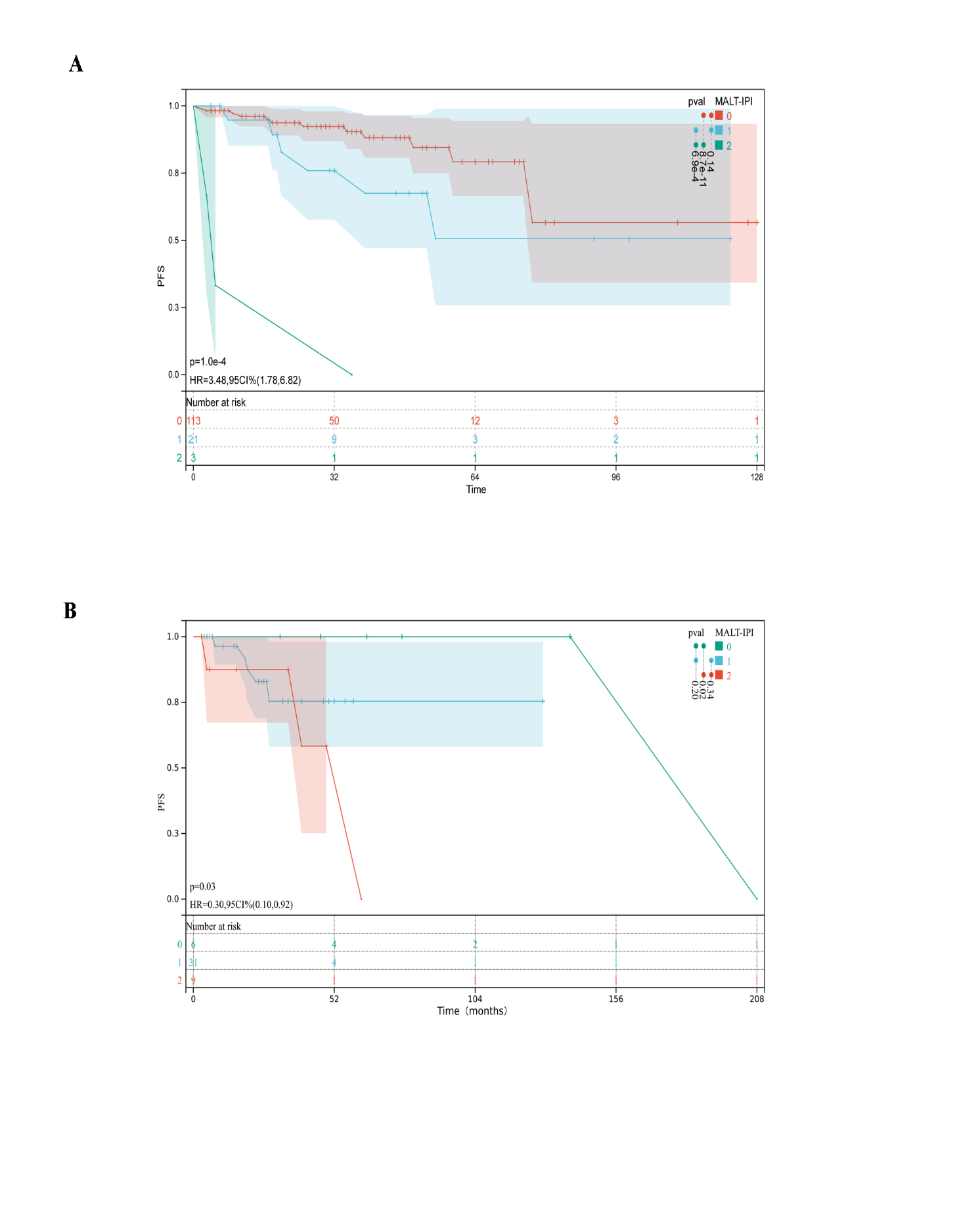


**Supplementary Figure 1.** Progression- free survival (PFS) for patients with MALT lymphoma were risk-stratified by MALT-IPI, and Kaplan Meier curves were performed. (A) Training cohort patients. (B) Validation cohort patients.
